# Primary mediastinal hydatid disease leading to popliteal artery hydatid cyst embolization

**DOI:** 10.4103/0256-4947.55174

**Published:** 2009

**Authors:** Aijaz Hakeem, Hakim Shafi, Tariq A. Gojwari, Shubana Rasool, Muneer Ahmad

**Affiliations:** a From the Department of Radiodiagnosis, SK Institute of Medical Sciences, Srinagar, Kashmir, India; b From the Department of Medicine, SK Institute of Medical Sciences, Srinagar, Kashmir, India; c From the Department of Surgery, SK Institute of Medical Sciences, Srinagar, Kashmir, India; d From the Department of Medicine, Government Medical College, Srinagar, Kashmir, India

## Abstract

Hydatid disease is a zoonosis caused by *Echinococcus granulosus*. Infected dogs release eggs through their feces and the eggs infect humans through food and water. The most common locations of hydatid cysts are the liver and lungs, but primary mediastinal involvement, though rare, can be encountered. We report on a 16-year-old female with a primary mediastinal hydatid cyst leading to popliteal arterial embolization. The mediastinal lesion was treated with partial pericystectomy with removal of the germinal membrane and prophylactic albendazole. In endemic areas, it is important to consider hydatid cysts in the differential diagnosis of an acute arterial occlusion.

Hydatid disease is an endemic disease in various geographical areas of world. The disease can involve almost every organ, though the liver and lung are common sites. Mediastial hydatid cysts, though rare, have been reported in literature.^1^,^2^ These hydatid cysts can also result in arterial occlusions in the aorta, iliac arteries, femoral arteries, popliteal arteries and even the mycardial arteries.^3^,^4^ We report a case of a primary mediastinal hydatid cyst in an adolescent leading to popliteal arterial embolization, in which the patient presented with a popliteal arterial hydatid cyst embolus. The primary cyst was seen on computed tomography in the mediastinum, eroding the descending thoracic aorta and an embolus with evidence of hydatid cyst componenets was found in the popliteal artery.

## CASE

**Figure 1:**
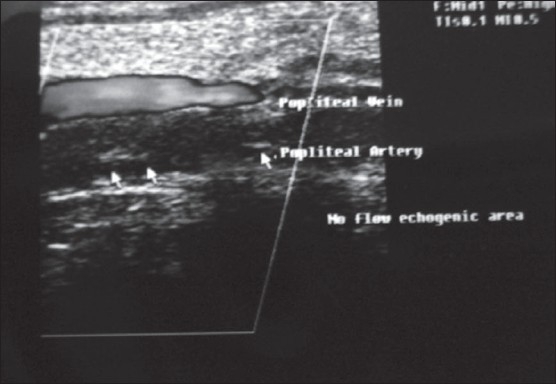
Popliteal artery embolus with linear echoic area within, with normal flow in the popliteal vein.

**Figure 2:**
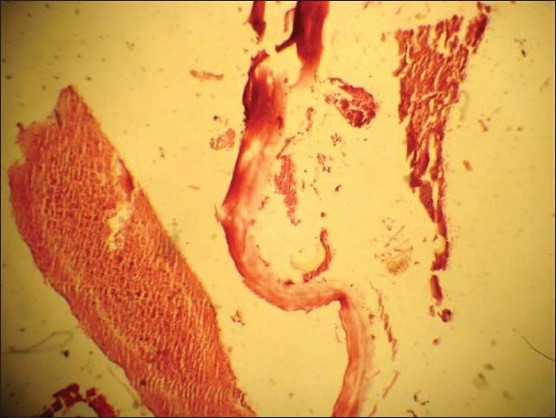
Photomicrograph showing laminated membrane of the hydatid cyst in an embolus.

**Figure 3:**
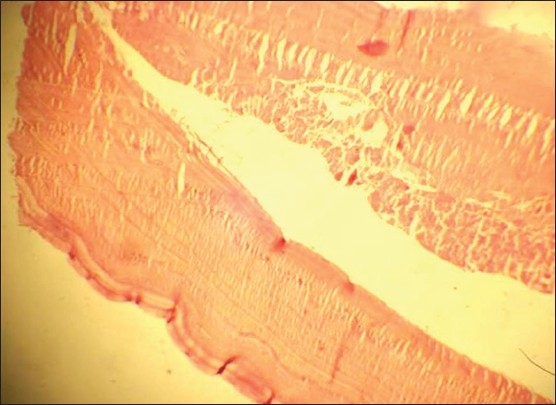
Another photomicrograph showing laminated membrane of the hydatid cyst in an embolus.

**Figure 4:**
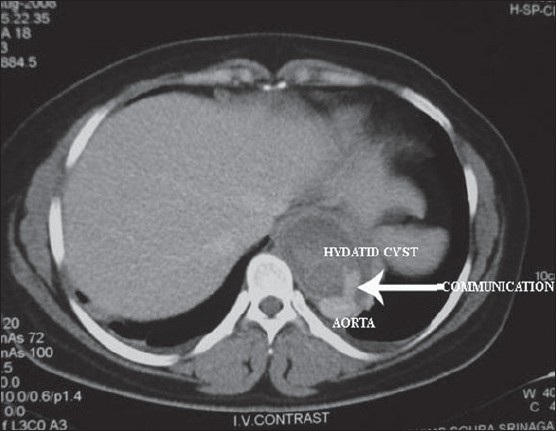
Transaxial contrast-enhanced multislice image showing posterior mediastinal hydatid cyst communicating with the aorta.

A 16-year-old Kashmiri female presented with pain in the left leg for 2 days. The pain was continuous in coarse and initially was localized to the lower calf and foot and then involved the whole leg. She had not had these symptoms before and had been previously healthy. On examination she had a pulse of 80/minute with bodytemperature of 98.5°F. The left leg was tender and cold with absent dorsalis pedis and posterior tibial arterial pulses on palpation. Homans sign was negative. There was no sensory loss. Joint movements and deep tendon reflexes were normal. Her abdominal and chest examinations were unremarkable. Doppler ultrasonography showed a hypoechoic popliteal arterial embolus with echogenic linear areas (Figure 1). A membrane-like structure was removed by urgent catheter embolectomy. A provisional diagnosis of intravascular hydatid cyst, leading to embolization was made, which was confirmed on histopathology (Figures 2 and 3). Serum serology for hydatid disease by ELISA test was negative. Abdominal ultrasonography was unremarkable. Chest x-ray showed a small left-sided pleural effusion. A multislice CT scan of the chest showed a cystic laminated structure in relation to the wall of the aorta, primarily arising from the mediastinum and eroding the wall of the aorta. This lesion appeared to be communicating with the aorta (Figure 4). No other abnormality was detected in the abdomen or chest on CT scan. Partial pericystectomy with removal of the germinal membrane was done through a median sternotomy approach and the patient was placed on prophylactic albendazole to avoid recurrence. Postoperative period was uneventful and the patient was discharged after 10 days.

## DISCUSSION

Hydatid disease is still a health problem in endemic areas of the world, especially in developing countries.^5^ The infection known as hydatidosis is a zoonotic infection caused by *Echinococcus granulosus* belonging to the class Cestoda. The organism lives in the small intestine of definitive hosts such as canines.^6^ Sheep and cattle are intermediate hosts and the echinococcal eggs enter the human body through food and water contaminated by the feces of infected canines. Children are not spared by this disease.^7^ Due to unhygienic conditions in developing countries the chances of getting hydatidosis are greater and this disease can involve any organ. Though the most common involved organs are the liver and lungs, hydatid cysts are seen also in other parts of body.^8^ The extrapulmonary and mediastinal involvement of hydatid cysts is rare.^9^,^10^ The most common location in the mediastinum is the posterior mediastinum.^11^ In one study of 1619 intrathoracic hydatid cysts, only 8 (0.5%) were found in the mediastinum.^12^ Complications caused by hydatid cysts if vascular invasion develops include anaphylactic shock, hemorrhage, systemic emboli and arterial occlusion.^13^ Marti-Bonmati et al documented a case on CT scan in which a hydatid cyst had ruptured into the aorta leading to bilateral popliteal embolism.^14^

The contrast-enhanced computed tomographic features represent a pathognomonic sign of a communicating rupture of an echinococcal cyst into the aorta.^14^ This case strongly suggest that in endemic areas for hydatid disease, it is important to consider hydatid cysts in the differential diagnosis of acute arterial occlusion. Screening should be done to rule out hydatid cyst in other parts of body, especially in relation to large vessels and when there is no clear cause or risk factor for arterial occulsion such as in this case.
